# Treatment of Active Crohn’s Disease With Exclusive Enteral Nutrition Diminishes the Immunostimulatory Potential of Fecal Microbial Products

**DOI:** 10.1093/ibd/izae124

**Published:** 2024-07-09

**Authors:** Caroline Kerbiriou, Caitlin Dickson, Ben Nichols, Michael Logan, Anna Mascellani, Jaroslav Havlik, Richard K Russell, Richard Hansen, Simon Milling, Konstantinos Gerasimidis

**Affiliations:** Human Nutrition, School of Medicine, Dentistry and Nursing, Glasgow Royal Infirmary, University of Glasgow, Glasgow, United Kingdom; Human Nutrition, School of Medicine, Dentistry and Nursing, Glasgow Royal Infirmary, University of Glasgow, Glasgow, United Kingdom; Human Nutrition, School of Medicine, Dentistry and Nursing, Glasgow Royal Infirmary, University of Glasgow, Glasgow, United Kingdom; Human Nutrition, School of Medicine, Dentistry and Nursing, Glasgow Royal Infirmary, University of Glasgow, Glasgow, United Kingdom; Department of Food Science, Czech University of Life Sciences Prague, Prague, Czech Republic; Department of Food Science, Czech University of Life Sciences Prague, Prague, Czech Republic; Department of Paediatric Gastroenterology, Hepatology and Nutrition, Royal Hospital for Sick Children and Young People, Edinburgh, United Kingdom; Department of Paediatric Gastroenterology, Hepatology and Nutrition, Royal Hospital for Children, Glasgow, United Kingdom; Division of Clinical and Molecular Medicine, Department of Child Health, School of Medicine, University of Dundee, Dundee, United Kingdom; School of Infection and Immunity, University of Glasgow, Glasgow, United Kingdom; Human Nutrition, School of Medicine, Dentistry and Nursing, Glasgow Royal Infirmary, University of Glasgow, Glasgow, United Kingdom

**Keywords:** Crohn’s disease, pediatric, exclusive enteral nutrition, immunogenicity, microbiota, metabolomics

## Abstract

**Background:**

Exclusive enteral nutrition (EEN) is an effective treatment for active Crohn’s disease (CD). This study explored the immunostimulatory potential of a cell-free fecal filtrate and related this with changes in the fecal microbiota and metabolites in children with active CD undertaking treatment with EEN.

**Methods:**

Production of tumor necrosis factor α (TNFα) from peripheral blood mononuclear cells was measured following their stimulation with cell-free fecal slurries from children with CD, before, during, and at completion of EEN. The metabolomic profile of the feces used was quantified using proton nuclear magnetic resonance and their microbiota composition with 16S ribosomal RNA sequencing.

**Results:**

Following treatment with EEN, 8 (72%) of 11 patients demonstrated a reduction in fecal calprotectin (FC) >50% and were subsequently labeled FC responders. In this subgroup, TNFα production from peripheral blood mononuclear cells was reduced during EEN (*P =* .008) and reached levels like healthy control subjects. In parallel to these changes, the fecal concentrations of acetate, butyrate, propionate, choline, and uracil significantly decreased in FC responders, and *p*-cresol significantly increased. At EEN completion, TNFα production from peripheral blood mononuclear cells was positively correlated with butyrate (rho = 0.70; *P =* .016). Microbiota structure (β diversity) was influenced by EEN treatment, and a total of 28 microbial taxa changed significantly in fecal calprotectin responders. At EEN completion, TNFα production positively correlated with the abundance of fiber fermenters from *Lachnospiraceae_UCG-004* and *Faecalibacterium prausnitzii* and negatively with *Hungatella* and *Eisenbergiella tayi*.

**Conclusions:**

This study offers proof-of concept data to suggest that the efficacy of EEN may result from modulation of diet-dependent microbes and their products that cause inflammation in patients with CD.

**Key messages**

**What is already known?**

The mechanism of action of exclusive enteral nutrition (EEN) used for the treatment of active Crohn’s disease is currently unclear but has previously been associated with extensive changes in the gut microbiota.

**What is new here?**

This study provides evidence that, in vitro, treatment of active Crohn’s disease with EEN leads to fecal microbial components with reduced proinflammatory potential, possibly resulting in a lower activation of host immune responses and amelioration of gut inflammation.

**How can this study help patient care?**

Assessing the proinflammatory potential of the gut microbiota and its products might help us predict patients who are more likely to demonstrate a response to EEN and optimize, prior to treatment initiation, those who are less likely to do so.

## Introduction

The commonly accepted cause of Crohn’s disease (CD) is an aberrant reaction of the gut immune system to the gut microbiota, in genetically predisposed individuals, under the influence of environmental factors.^1^ The way specific microbes and their metabolites interact with the host’s immune system initiating gut inflammation and how established therapies abrogate this effect remain largely unknown. Unraveling these complex relationships is of crucial importance in improving disease management options and progression toward cure.

Exclusive enteral nutrition (EEN) is used for the treatment of active CD, improving biomarkers of gut inflammation and inducing mucosal healing.^2-5^ The mode of action of EEN is currently unclear but probably involves the exclusion of dietary components from host diet that interact with or act as substrate for inflammatory components of the microbial community.^6^ In previous research, improvement of clinical disease activity and normalization of inflammatory biomarkers, during treatment with EEN, were associated with extensive changes in the gut microbiota.^7-9^ Such changes included a decline in pathobionts and fiber-fermenting organisms, alongside changes in the fecal metabolome with a notable decrease in the levels of short-chain fatty acids (SCFAs).^10^ Whether these EEN-induced changes in the microbiota and its function are core mechanisms of action of the therapy or simply secondary epiphenomena of dietary modification and treatment need to be addressed within specific mechanistic investigations.

This proof-of-concept study assessed the effect of treatment with EEN on the immunostimulatory potential of microbial products in cell-free fecal filtrates of children with CD, via stimulation of tumor necrosis factor α (TNFα) secretion from peripheral blood mononuclear cells (PBMCs) and integrated analyses with the fecal metabolome and microbiota composition.

## Methods

### Patients, Fecal Sample Collection, and Cell-Free Fecal Filtrates

Participants were recruited from the Royal Hospital for Children in Glasgow. Eligible participants were pediatric patients with active CD initiating treatment with EEN as standard of care. Participants were selected from a larger cohort, previously described in the in the literature.^3^ Selection of patients was based on complete sample availability and measurements of fecal calprotectin (FC) at each time point of the study observational period. Sex- and age-matched healthy control (HC) subjects were recruited from the local community.

In total, 11 participants with CD and 11 HC subjects were included (participants with CD mean age 12.7 ± 2.9 years vs HC subjects mean age 10.4 ± 3 years). All patients with CD were treated with Modulen IBD (Nestlé Health Science), a casein-based polymeric formula enriched with transforming growth factor β, for 8 weeks. Fresh fecal samples were collected and processed within 2 hours of passage, prior to initiation of EEN, at 4 and 8 weeks of treatment. Patients in whom FC decreased >50% during EEN were classed as FC responders; those with a <50% decrease in FC were classed as FC nonresponders. A 50% decrease in FC was selected as a cutoff, as this has previously been shown to be a predictive factor for inactive endoscopic disease in a pediatric patients with CD on EEN.^11^

Fecal slurries (0.1%) were prepared by suspending 10 mg of fecal samples to 10 mL of sterile distilled water. The slurries were homogenized for 5 minutes with the help of glass beads (3 mm) on an orbital shaker (VXR Basic, IKA) at 1200 rpm. Following a 5-minute centrifugation at 12 000 *g*, the supernatant was recovered and filtered through 0.22 μm to produce a cell-free filtrate. The cell-free fecal filtrate was stored at −20 °C until further use.

### PBMC Isolation and Stimulation

Blood samples were collected from healthy adult volunteers. A total of 4 mL of fresh blood were layered onto 4 mL of Histopaque-1077 (Sigma-Aldrich) and centrifuged for 20 minutes at 400 *g* at 18 °C. The PBMCs were subsequently isolated and washed 3 times with 1 ml of 1X Dulbecco’s phosphate-buffered saline (Gibco). PBMCs were suspended in serum/free RPMI 1640 Medium (Gibco) containing 1% L-glutamine solution (Sigma-Aldrich) and 1% penicillin-streptomycin (Sigma-Aldrich, Darmstadt, Germany) and counted in a 1:1 (v:v) ratio of trypan blue (Gibco) to assess viability. PBMCs were seeded on 24-well sterile plates (Corning) at 0.5 million cells per well, contained in 400 µL of media. PBMCs were stimulated by adding 100 µL of 0.1% cell-free fecal filtrate. After 24 hours of incubation, the supernatants were recovered and stored at −80 °C. PBMCs were dissociated by adding 0.05% Trypsin-EDTA (Gibco) and counted on a hemocytometer with trypan blue (Gibco) to ensure cell viability >90%. Validation data did not show differences in response throughout the EEN treatment when the same fecal slurries were used to stimulate PBMCs from different blood donors (data not shown). However, all samples from the same patient were incubated with the same PBMC collected from a single blood donor, and a matched HC subject was also included to minimize interassay variation in comparative analysis between the 2 participant groups.

### Quantification of FC, TNFα, and Gluten Immunogenic Peptides

The secretion of TNFα from stimulated PBMCs was measured with the Human TNF-α uncoated ELISA kit (Invitrogen). The concentration of calprotectin in the fecal samples of the patients was assessed with the CalproLAB ELISA kit (Svar). Gluten immunogenic peptides were quantified using the iVYDAL In Vitro Diagnostics iVYLISA GIP-S kit (Biomedal S L). Detection of GIP in feces collected during EEN (which does not contain gluten) was deemed as a proxy biomarker of nonexclusive compliance to treatment.^12^

### Metabolite Concentration Using Nuclear Magnetic Resonance

Proton nuclear magnetic resonance (^1^H NMR) was used to characterize the fecal metabolome of patients with CD and healthy children in feces. Briefly, fecal samples were diluted in phosphate-buffered saline buffer (200 mg/mL), sonicated for 5 minutes, and vortexed prior to further processing. A total of 1 mL of fecal slurry was centrifuged at 16 000 *g* for 15 minutes at 4 °C. After centrifugation, 70 µL of NMR solution buffer (1.5 M K_2_HPO_4_ + 1.5 M Na_2_HPO_4_, pH 7.4, 0.2% NaN_3_, 5 mM TSP) was added to 630 µL of supernatant, incubated at room temperature for 10 minutes, and centrifuged again at 24,400 *g* for 15 minutes at 4 °C. A total of 600 µL of this mixture was transferred to a 5 mm standard ^1^H NMR tube. All the ^1^H NMR spectra were acquired using a Bruker AVANCE III HD 500 MHz spectrometer equipped with a 5-mm BBO probe operating at 500.23 MHz. All measurements were performed at 298 K. The Bruker pulse sequence ʺnoesypr1dʺ with suppression of water signal at 4.704 ppm was used. ^1^H NMR experiments were acquired at time domain of 64 k real data point using a 16 ppm spectral width, 128 scans and 2 dummy scans, a relaxation delay of 1 second, acquisition time of 4 seconds, and 100 ms of mixing time. Tuning and matching, locking, shimming, and pulse calibration were automatically done using standard module routines (atma, lock and topshim 3D, and pulsecal AU program). ^1^H NMR spectral data were processed by Fourier transformation, calibration to TSP at 0.0 ppm, and manual phase in TopSpin 3.6.4 (Bruker). Spectra were further processed in Chenomx NMR suite 8.4 software, to identify and quantify metabolites. The metabolite signature database used was Chenomx Reference Library 500 MHz version 10. Concentrations were expressed as μmol/g of wet fecal sample.

### Gut Microbiota Composition and Bioinformatics

Bacterial DNA was extracted using the QIAGEN DNeasy Powersoil pro DNA kit according to manufacturer’s instructions and DNA extracts were sent to the NU-OMICS DNA sequencing research facility at the University of Northumbria for sequencing of the V4 region of 16S ribosomal RNA.

The gut microbiome was represented using an amplicon sequence variant (ASV) table, generated using DADA2 pipeline version 1.16.^13^ This process first involved quality filtering of the raw sequencing output, whereby reads with an expected error rate >2 were removed and those remaining were truncated at the first instance of an Illumina quality score <2. The main DADA2 algorithm was then applied, using machine learning to infer sequence variants based on error rates. Paired-end reads were merged to form ASVs. Chimeras were then removed from the data using the DADA2 de novo chimera detection method. Taxonomic information, up to genus level, was assigned to each ASV using the RDP Naive Bayesian Classifier algorithm in conjunction with the SILVA 138 reference database.^14^ Species-level assignments were also added to those ASVs that matched exactly with the reference database. Statistical analysis was performed using R version 4.2.3 (R Foundation for Statistical Computing) with functions from published R packages in conjunction with scripts that were coded in-house specifically for use with this dataset. Data were explored in terms of α diversity using 4 metrics: Chao1 richness estimate, Shannon diversity index, and Pielou’s evenness. Mann-Whitney tests were used to assess significance between study timepoints. Overall community composition was visualized using nonmetric multidimensional scaling of Bray-Curtis dissimilarity matrices and evaluated using permutation analysis of variance. Differences in abundances of individual ASVs between timepoints were identified using paired Mann-Whitney tests with multiple testing corrected for using the Benjamini-Hochberg method, and a level of significance set at *P <* .15 for adjusted *P* values.

### Statistical Analysis

Differences in FC, TNFα produced from stimulated PBMCs, and fecal metabolite concentrations during EEN were tested with a general linear model with participant identification number added as a random factor. A 2-sample *t* test was used to assess significance between the HC and CD groups. Correlations between the concentration of TNFα produced from stimulated PBMCs with fecal metabolites was tested using Spearman rank correlation at all timepoints (EEN start, 33 days of EEN, and 54 days EEN), at individual time points for the CD patients, and at the single collection point for the HC subjects. The Spearman test was used to evaluate correlations between microbial ASVs, FC, TNFα, and other relevant fecal metabolites. Where multiple tests were carried out, *P* values were corrected using the Benjamini-Hochberg method with a level of significance set at *P <* .15 for adjusted *P* values. All statistical analysis were completed on Minitab v20.3 or with R version 4.2.3 (R Foundation for Statistical Computing).

### Ethical Considerations

The study was approved by the NHS West of Scotland Research Ethics Committee (14/WS/1004) and was registered at ClinicalTrials.gov (NCT02341248).

## Results

### Changes in FC During Treatment With EEN

**Figure 1.**
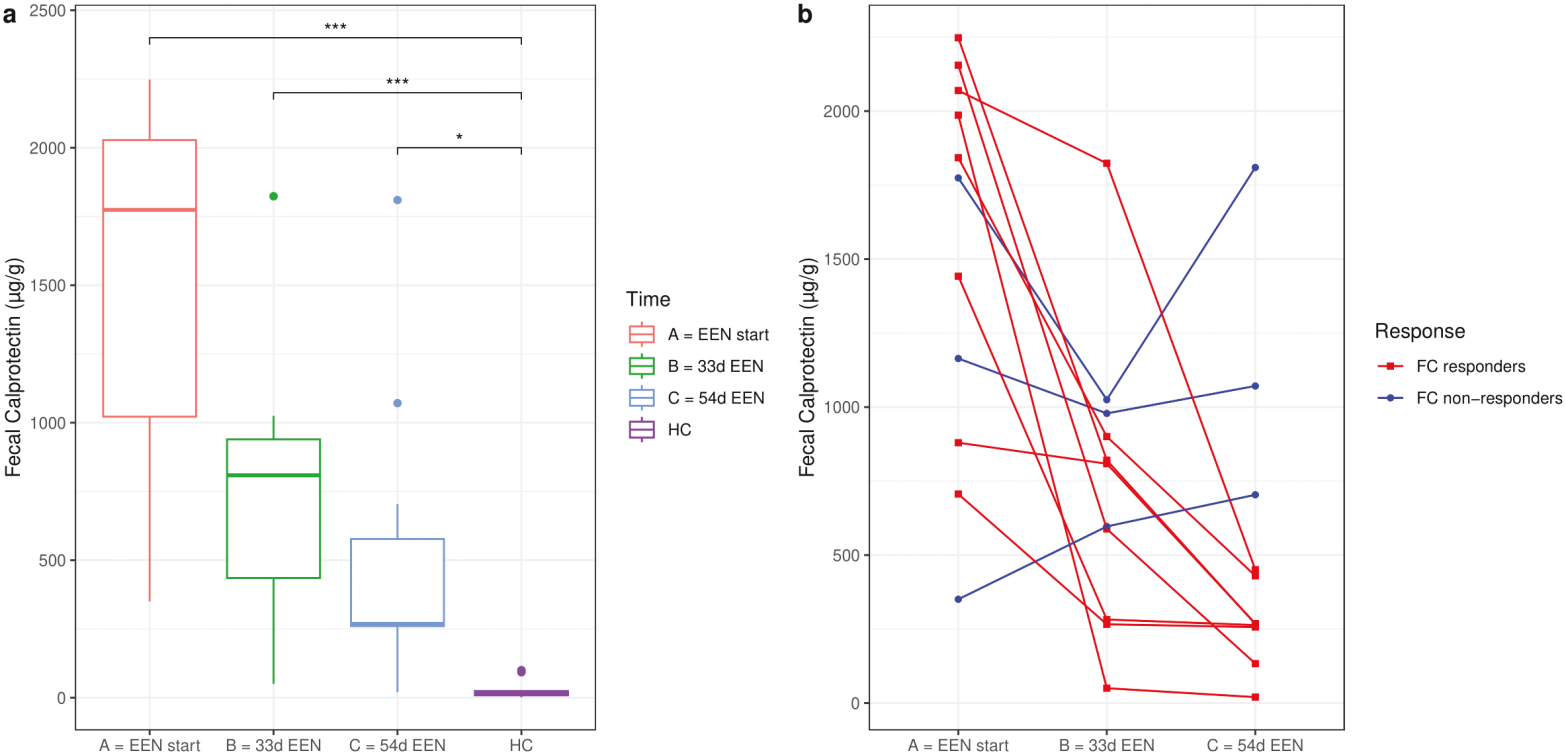
Fecal calprotectin (FC) in Crohn’s disease (CD) patients and healthy control (HC) subjects. (A) FC in CD patients (n = 11) at start of exclusive enteral nutrition (EEN) (red), after 33 days on EEN (green), and after 54 days on EEN (blue), and in HC subjects (n = 11; purple). (B) Individual changes in FC in CD patients (n = 11) classified as FC responders (n = 8; red; drop ≥50% in FC during EEN) or FC nonresponders (n = 3; blue; drop ≥50% in FC during EEN). *P < .05; **P < .01; ***P < .001.

Participants’ characteristics are presented in Table 1. Average FC levels significantly decreased during EEN (*P* = .02 for baseline vs 33 days and *P* < .001 from baseline vs 54 days of EEN) (Figure 1A). At EEN completion, 8 (72%) out of 11 patients presented a decrease in FC of more than 50% (median 80.0% [interquartile range, 71.3%-92.4%]) and were labeled as FC responders (Figure 1BSupplementary Table 1). All FC responders had an FC <500 mg/kg at EEN completion (Figure 1B). Ten (91%) out of 11 patients achieved clinical remission at EEN completion (weighted pediatric Crohn’s Disease Activity Index < 12.5). All 8 patients classified as FC responders had also achieved clinical remission at EEN completion (Table 1). Two patients (1 responder and 1 nonresponder) had detectable gluten in feces, indicating poor compliance to EEN.

**Table 1.** Participants characteristics.

	CD patients	Healthy control subjects
n	11	11
Female/male^a^	5/6	4/7
Age, y^a^	13.3 (9.9 to 15.0)	10.8 (7.0 to 13.0)
Height z-score^a^	−0.2 (−0.9 to 0.7)	0.7 (−0.9 to 1.0)
Weight z-score^a^	−0.6 (−0.9 to −0.1)	0.2 (−0.9 to 1.0)
BMI z-score^a^	−0.2 (−1.7 to 0.2)	−0.3 (−0.9 to 1.2)
Disease location		
Ileal	0	N/A
Ileocolonic	9	N/A
Colonic	2	N/A
wPCDAI (baseline)	45 (27.5 to 57.5)	N/A
wPCDAI (end of EEN)	0 (0 to 0)	N/A

Values are n or median (interquartile range).

Abbreviations: BMI, body mass index; CD, Crohn’s disease; N/A, nonapplicable; wPCDAI, weighted Pediatric Crohn’s Disease Activity Index.

^a^No significant differences observed between the CD patients and the healthy control subjects (t test; *P* > .05).

### TNFα Production From PBMCs Exposed to Fecal Immunostimulatory Microbial Products of Children With CD During EEN

**Figure 2.**
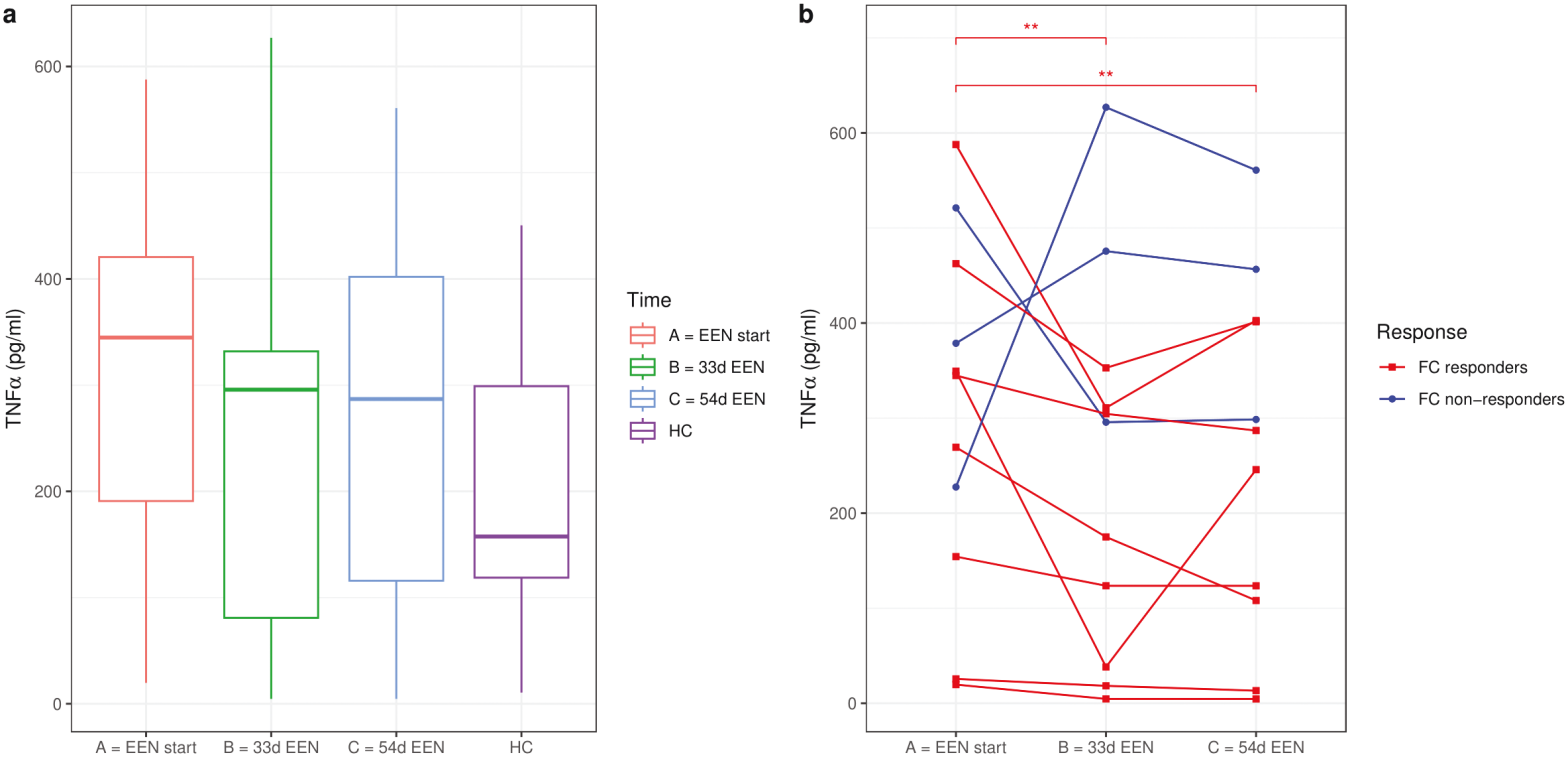
Tumor necrosis factor α (TNFα) concentration secreted by peripheral blood mononuclear cells stimulated with cell-free fecal filtrates of Crohn’s disease patients for 24 hours. (A) TNFα concentration (pg/mL) secreted by peripheral blood mononuclear cells stimulated with cell-free fecal filtrations of Crohn’s disease patients (n = 11) at start of exclusive enteral nutrition (EEN) (red), after 33 days on EEN (green), and after 54 days on EEN (blue), and in healthy control (HC) subjects (n = 11; purple). (B) Individual variations of TNFα concentration (pg/mL) secreted by peripheral blood mononuclear cells stimulated with cell-free fecal filtrates of fecal calprotectin (FC) responders (n = 8; blue) and FC nonresponders (n = 3; red) collected at the start of EEN and after 33 days and 54 days on EEN, and the fecal slurries of HC subjects (black). **P = .008 compared with EEN start for FC responders only.

Stimulation of PBMCs with cell-free fecal filtrates of CD patients collected prior to EEN led to a comparable secretion of TNFα to that provoked by filtrates from HC subjects, albeit the median concentration of TNFα only tended (*P =* .138) to be higher in patients with CD than HC subjects. Similar observations were obtained with fecal filtrates collected at 33 and 54 days of EEN and compared with the HC subjects (*P =* .484 and *P =* .337, respectively) (Figure 2A).

As a group, secretion of TNFα from PBMCs did not change in response to stimulation with cell-free fecal filtrates from patients with CD during EEN (*P =* .458) (Figure 2A). A positive correlation was observed in TNFα production between the start and the end of EEN (rho = 0.655; *P =* .029). However, in subgroup analysis of FC responders only, a statistically significant decrease in TNFα secretion was observed when PBMCs were stimulated with cell-free fecal slurries collected after 33 days (*P =* .008) and 54 days (*P =* .008) of EEN compared with initiation of EEN (Figure 2B). Although TNFα production did not differ between FC responders and nonresponders, the latter group presented higher levels throughout treatment, particularly at EEN completion (*P =* .052) (Supplementary Figure 1). Changes (fold change) in FC levels did not correlate with changes in the production of TNFα from PBMCs during treatment with EEN (Supplementary Figure 2).

### Changes in Fecal Metabolome During Treatment With EEN in CD Patients and in Relation to TNFα Production From PBMCs

**Figure 3.**
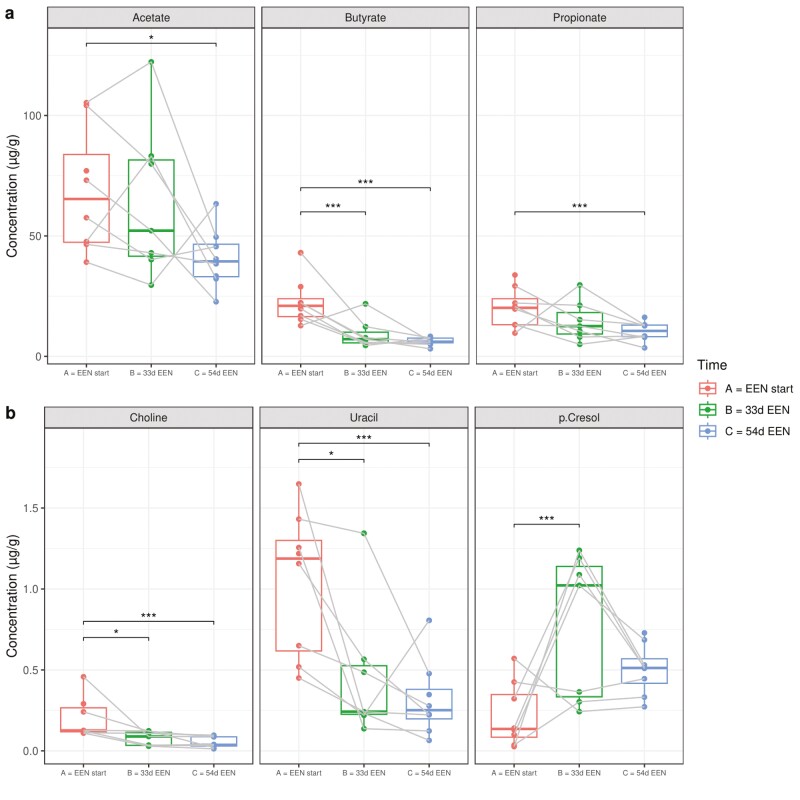
Fecal metabolites significantly influenced by treatment with exclusive enteral nutrition (EEN) in fecal calprotectin responders. Fecal concentrations (µg/g) of (A) short-chain fatty acids acetate, butyrate, and propionate and (B) choline, uracil, and *p*-cresol in fecal calprotectin responders at the start of EEN (red), after 33 days on EEN (green) and after 54 days on EEN (blue). *P < .05; ***P < .001.

A total of 29 metabolites were annotated and quantified with ^1^H NMR. The concentrations of 6 metabolites significantly changed from baseline within 33 and 54 days on EEN treatment in CD patients identified as FC responders. These included the concentration of acetate, butyrate, propionate, choline, and uracil, which all significantly decreased in these patients, while that of *p*-cresol increased after 33 days on EEN (Figure 3). Similar results were observed in the entire CD cohort (Supplementary Figure 3).

Correlations between fecal metabolites impacted by EEN treatment in the FC responders and TNFα produced by stimulated PBMCs were investigated in the entire CD cohort (n = 11). There were no linear relationships between the fold changes of metabolites and those of TNFα production during treatment with EEN (Supplementary Figures 4 and 5). However, at EEN treatment completion, the concentration of secreted TNFα was positively correlated with butyrate (rho = 0.70; *P =* .016). No other significant correlations were observed at study initiation, at 33 days on EEN, or at treatment completion. Likewise, there were no statistically significant correlations between TNFα concentrations with any of the metabolites measured in samples taken from HC subjects.

### Changes in Fecal Microbiota During Treatment With EEN in CD Patients and in Relation to TNFα Production From PBMCs

**Figure 4.**
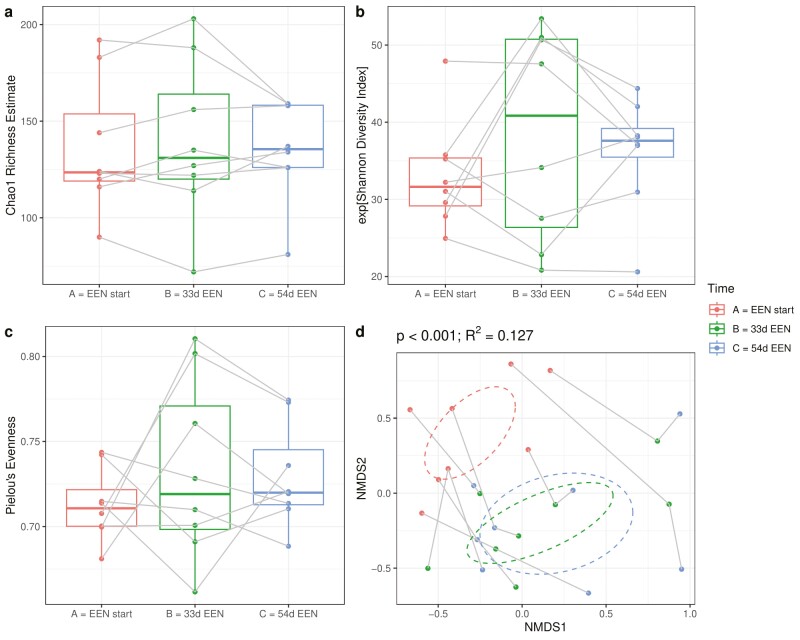
Fecal microbial α diversity and β diversity in fecal calprotectin responders prior and during treatment with exclusive enteral nutrition (EEN). (A) Chao1 richness estimate, (B) Shannon diversity index, (C) Pielou’s evenness, and (D) nonmetric multidimensional scaling plot in FC responders (n = 8) prior to treatment (red), at 33 days on EEN (green), and at 54 days on EEN (blue).

Microbiota data were obtained from all 33 samples of all 11 CD patients at the start of EEN, after 33 days on EEN, and after 54 days on EEN. Microbiota α diversity indices were similar between the 3 sample collection points. In contrast, the microbiota structure (β diversity) was significantly influenced by treatment, and it was significantly different after 33 days (R^2^ = 0.122) and 54 days (R^2^ = 0.100) on EEN (both *P <* .001) (Supplementary Figure 6). No differences were observed between the 2 successive samples collected during EEN (*P =* .509). Similar microbial signals were obtained also for the subgroup of FC responders for α diversity and β diversity, albeit the microbial effects were much more pronounced in the FC responders, despite a smaller sample size (EEN start vs 33 days EEN: R^2^ = 0.137, *P =* .012; EEN start vs 54 days EEN: R^2^ = 0.136, *P =* .012; 33 days EEN vs 54 days EEN: R^2^ = 0.017, *P =* .547) (Figure 4).

**Figure 5.**
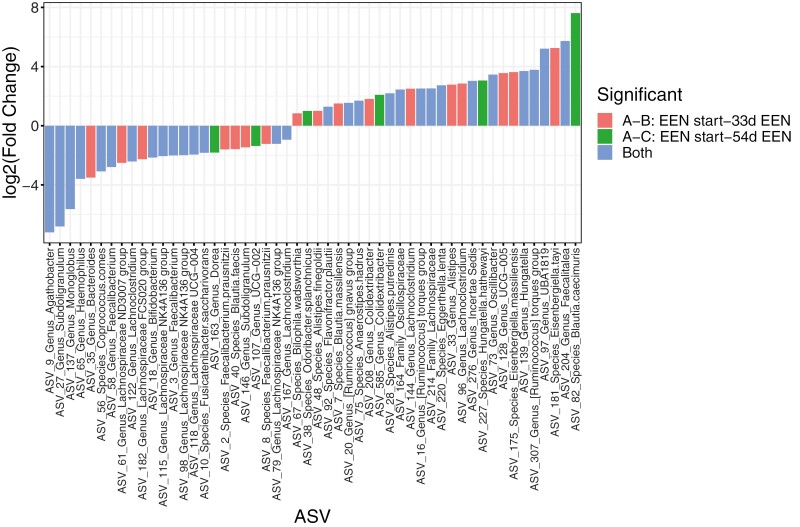
Fold change of differential amplicon sequence variants (ASVs) in all Crohn’s disease patients (n = 11) over the course of exclusive enteral nutrition (EEN) treatment. Log2 fold change of microbial ASVs presenting differences in relative abundance at 33 days of treatment (red), at 54 days on EEN (green), or both (blue) compared with the start of EEN treatment in all Crohn’s disease patients. For the ASVs that were different at both time points, we display the log2 fold change of the difference between EEN start and 54 days on EEN.

A total of 46 microbial ASVs significantly differed between the start of EEN and 33 days into treatment (adjusted *P* < .15) (Figure 5Supplementary Table 2). The abundance of 22 ASVs decreased after 33 days on EEN, notably *Subdoligranulum*, *Haemophilus*, *Agathobacter*, *Monoglobus*, *Coprococcus comes*, *Lachnospiraceae NK4A136 group*, *Lachnospiraceae_UCG-004*, and *Faecalibacterium.* Fifteen (68%) of these ASVs remained at significantly lower abundance in the fecal samples at 54 days of treatment, with the addition of *Bifidobacterium* (Figure 5Supplementary Table 3). In contrast, after 33 days on EEN, the abundance of 24 ASVs including *Faecalitalea*, *UBA1819*, *Oscillibacter*, *Hungatella*, *Incertae Sedis*, *Lachnoclostridium*, *Alistipes*, and *Ruminococcus torques group* increased, 14 (58%) of which remained at higher abundance at the point of EEN completion and compared with baseline (Supplementary Tables 2 and 3).

**Figure 6.**
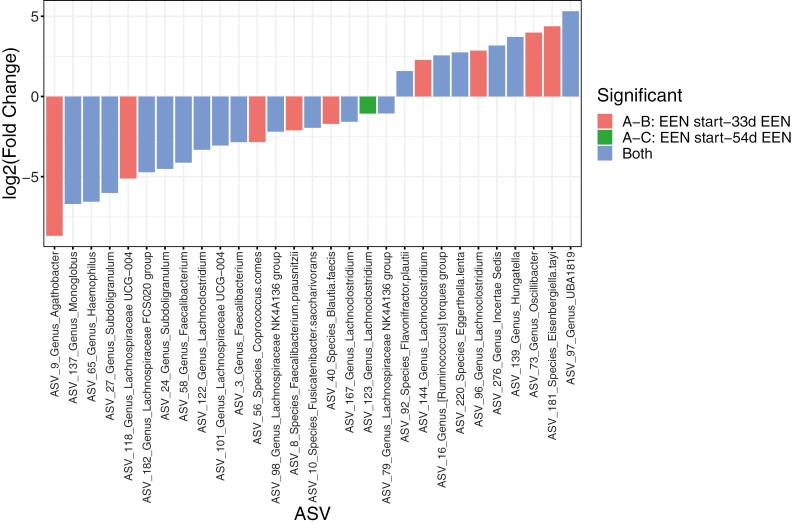
Fold change of differential amplicon sequence variants (ASVs) in fecal calprotectin responders (n = 8) over the course of exclusive enteral nutrition (EEN) treatment. Log2 fold change of microbial ASVs presenting differences in relative abundance at 33 days of treatment (red), 54 days on EEN (green), or both (blue) compared with the start of EEN treatment in fecal calprotectin responders. For the ASVs that were different at both time points, we display the log2 fold change of the difference between EEN start and 54 days on EEN.

Similar analysis in the subgroup of FC responders showed that the relative abundance of 28 ASVs differed between the start of treatment and 33 days on EEN (Figure 6Supplementary Table 4,), 19 (68%) of which remained statistically different after 54 days of treatment (Figure 6, Supplementary Table 5).

Correlations between the abundance of fecal ASV impacted by the EEN treatment in the FC responders and the concentration of TNFα produced by stimulated PBMCs were investigated in the entire CD cohort (n = 11). Like with fecal metabolites, there were no linear associations between the fold changes of ASVs and TNFα production from PBMCs during treatment with EEN.

**Figure 7.**
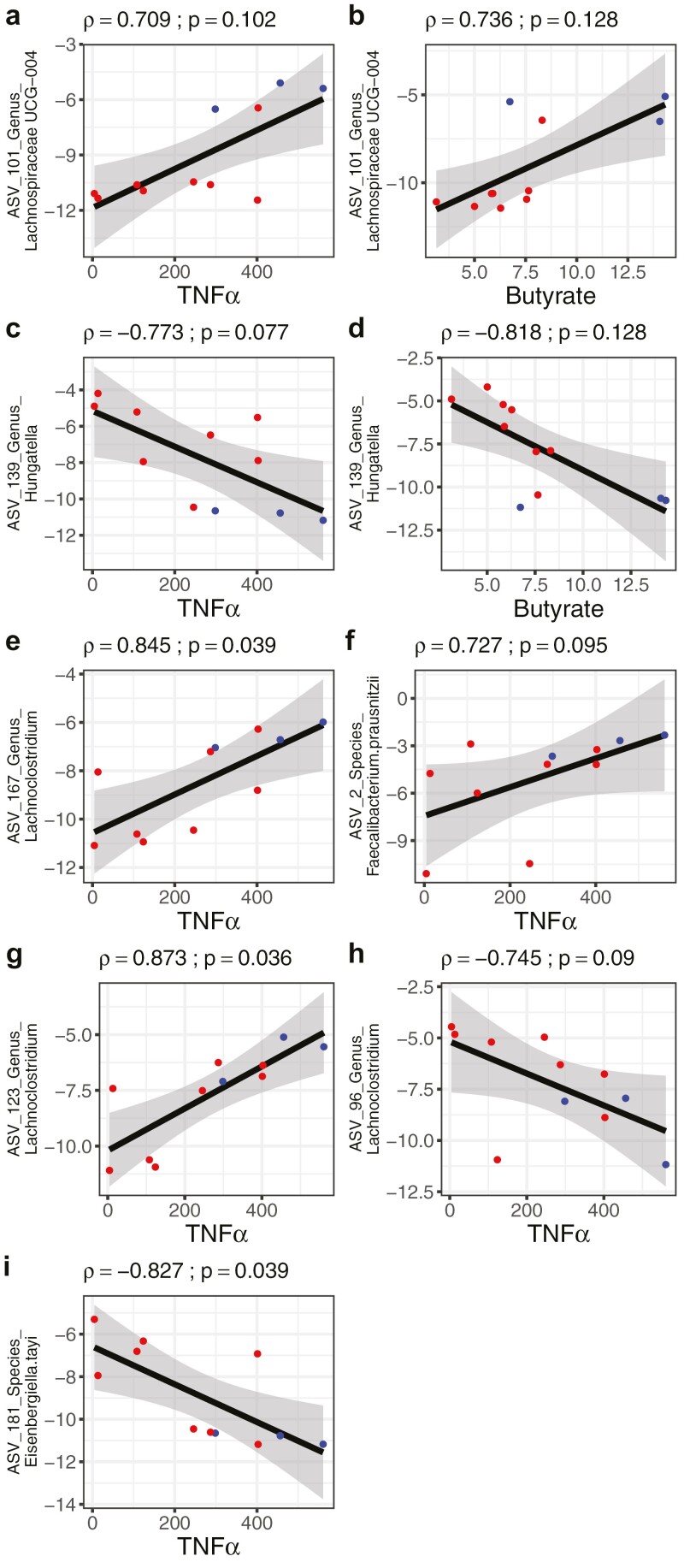
Correlation between microbial amplicon sequence variants, secreted tumor necrosis factor α (TNFα) and fecal metabolites in Crohn’s disease patients (n = 11) at 54 days on exclusive enteral nutrition (EEN) treatment completion. Spearman correlations between microbial amplicon sequence variants and TNFα (A, C, E-I) and butyrate (B, D) at 54 days of EEN. Fecal calprotectin responders (n = 8) are represented in red and FC nonresponders (n = 3) are represented in blue.

Last, we tested correlations between the abundance of taxa that changed significantly during EEN in the FC responders and the concentration of TNFα production from PBMCs at each time point of EEN. No significant correlations were observed at study initiation or after 33 days on EEN. At EEN completion (54 days on EEN), TNFα production from PBMCs was positively related with the relative abundance of ASV_101_Genus_Lachnospiraceae UCG-004 (rho = 0.71; *P =* .102); this same organism was also positively related with the concentration of butyrate (rho = 0.74; *P =* .128). In contrast, strong negative correlations were observed between TNFα production from PBMCs and the relative abundance of ASV_139_Genus_Hungatella (rho = −0.77; *P =* .077), with this organism also being negatively related with butyrate concentration (rho = −0.82; *P =* .128) (Figure 7, Supplementary Figure 6).

Positive strong associations were also observed between TNFα production from PBMCs and taxon abundance for ASV_167_Genus_Lachnoclostridium (rho = 0.85; *P =* .039), ASV_2_Species_Faecalibacterium.prausnitzii (rho = 0.73; *P =* .095), and ASV_123_Genus_Lachnoclostridium (rho = 0.87; *P =* .036). In contrast, negative associations were observed with ASV_96_Genus_Lachnoclostridium (rho = −0.75; *P =* .090) and ASV_181_Species_Eisenbergiella.tayi (rho = −0.83; *P =* .039). The abundance of several other organisms correlated with the levels of fecal metabolites measured but not with the extent of TNFα production (Supplementary Figure 7).

## Discussion

Here, we provide evidence that treatment of active CD with EEN leads to fecal microbial components with reduced proinflammatory potential, possibly resulting in a lower activation of host immune responses and amelioration of gut inflammation. We also observed that production of TNFα by PBMCs tended to be higher in response to the cell-free fecal slurry of treatment-naïve pediatric CD patients than HC subjects, and that this immunogenicity decreased during treatment with EEN and returned to similar levels as seen in HC subjects. More importantly, we observed that the cell-free fecal filtrates of patients who did not demonstrate an FC response to treatment with EEN tended to be more proinflammatory and stimulated a higher production of TNFα than those who did. Should these signals be replicated in larger studies, they could mean that stimulation of TNFα from PBMCs with cell-free fecal slurries might be an appropriate means to assess the proinflammatory potential of the gut microbiota and its products. In turn, this could help clinicians to predict, prior to treatment initiation, patients who might be more likely to demonstrate a response to EEN, hence moving toward the concept of stratified nutritional therapy.

Several bacterial metabolites and microbial taxa changed during treatment with EEN, and it is possible that these microbial signals are important contributors to the immunogenicity of the cell-free fecal filtrates we observed here. At treatment completion, butyrate, and fiber fermenters like *F prausnitzii*, *Lachnoclostridium*, and Lachnospiraceae were associated with proinflammatory potential, whereas the opposite was observed for *Hungatella* and *E tayi*, which associated with anti-inflammatory effects. However, we did not identify statistically significant linear relationships between changes in the levels of metabolites or microbial taxa and production of TNFα from PBMCs, demonstrating the complexities in identifying organisms and metabolites playing primary roles in provocation of gut inflammation in CD, and in unraveling the mechanism of EEN action. In health, fiber intake and fiber-originating SCFAs have been positively associated with anti-inflammatory effects, but in the presence of mucosal inflammation and damage, like in CD, detrimental effects have also been described. In primary epithelial monolayers of patients with UC and healthy control subjects, butyrate did not protect against inflammation-induced barrier dysfunction, and even worsened it.^15^ Similarly, recent research showed that unfermented β-fructan, a prebiotic fiber, exacerbated inflammation in certain patients with IBD, hence innocuous or even beneficial nutrients for human health may have harmful effects in patients with IBD, at least in a proportion of patients who respond to the dietary fiber-free EEN.^16^ These previous mechanistic data are also supported by clinical trials that confirmed, repeatedly, the ineffectiveness of fiber and prebiotic supplementation to improve disease outcomes in patients with active CD. According to these findings, it could be hypothesized that EEN promotes the reduction of the proinflammatory intestinal environment by modulating the gut microbiota, including via deprivation of fiber substrates for bacterial growth, with consequent reduced production of SCFAs.^6^

The current in vitro experiment used here presents several limitations. The TNFα response observed most likely resulted from the activation of the innate response as PBMCs were incubated with fecal slurries for a 24-hour period. It is likely that TNFα was secreted by monocytes as a result of the activation of the TLR-4 pathway^17^ and the use of nuclear factor kappa B inhibitors as control subjects could be beneficial to confirm the involvement of this pathway. The use of patients’ own intestinal cells or PBMCs might present a better model to study the systemic and local innate and adaptive responses to EEN, and corroborate findings reported here. The quantification of a larger panel of pro- and anti-inflammatory proteins would have allowed for a deeper profiling and understanding of the immune response mediated by microbiota-derived products before, during, and after EEN to replicate or refute our findings based on a single proinflammatory cytokine. Although 16S ribosomal RNA amplicon sequencing offers a global profiling of the gut microbiota, comprehensively profiling the composition of fecal microbial metabolites is far more complex, and there is no single analytical method to do so. It is therefore possible that other microbial products, including proteins, lipids, and unknown metabolites, induce the immunogenic effects observed here and that the relationship with specific metabolites that we observed are simply biomarkers of organisms producing them.^18^ Another limitation of the study is that the associations between the dietary intakes of HC subjects and fecal metabolites could not be explored, as dietary records were not collected. Furthermore, this study did not investigate the effect of reintroduction of an unrestricted diet, after completion of EEN, on the immunostimulatory potential of fecal microbial products, which could help identify compounds associated with maintenance or loss of remission.

## Conclusions

We observed that fecal microbial products from samples collected during EEN were less immunogenic than prior to treatment, and the secretion of proinflammatory protein TNFα positively correlates with products of fiber fermentation, like butyrate, which diminished during EEN alongside major fiber-fermenting organisms. Although a direct contribution of butyrate to the pathogenesis of CD is unclear, these findings offer proof-of concept data to suggest that the benefits of EEN may result from modulation of diet-dependent microbes, which otherwise cause inflammation in patients with CD. Future research should leverage the data presented here to explore whether selective manipulation of identified microbial species and metabolites can induce similar efficacy to EEN.

## Supplementary data

Supplementary data is available at *Inflammatory Bowel Diseases* online.

izae124_suppl_Supplementary_Tables_1-5_Figures_1-7
