# Supplementary material for: Treatment of Active Crohn’s Disease With Exclusive Enteral Nutrition Diminishes the Immunostimulatory Potential of Fecal Microbial Products
Source: Inflamm Bowel Dis. 2024 Jul 9;30(12):2457–66. doi: 10.1093/ibd/izae124 (PMC11630284; doi:10.1093/ibd/izae124)
Supplement: izae124_suppl_Supplementary_Tables_1-5_Figures_1-7 [file izae124_suppl_supplementary_tables_1-5_figures_1-7.docx]

**Supplementary table 1. Fecal calprotectin variations between the start of EEN and the end of EEN (54 days) in CD patients (n=11)**

| **Fecal calprotectin at the start of EEN (µg/g)** | **Fecal calprotectin after 54 days on EEN (µg/g)** | **Changes in fecal calprotectin during EEN (%)** | **FC responders (FC drop of ≥50%)** |
| --- | --- | --- | --- |
| 1843 | 430 | -77 | Yes |
| 1774 | 1810 | +2.0 | No |
| 880 | 268 | -69 | Yes |
| 1164 | 1071 | -8 | No |
| 1986 | 20 | -99 | Yes |
| 2155 | 133 | -94 | Yes |
| 2069 | 450 | -78 | Yes |
| 2248 | 267 | -88 | Yes |
| 1442 | 263 | -82 | Yes |
| 351 | 704 | +101 | No |
| 706 | 257 | -64 | Yes |


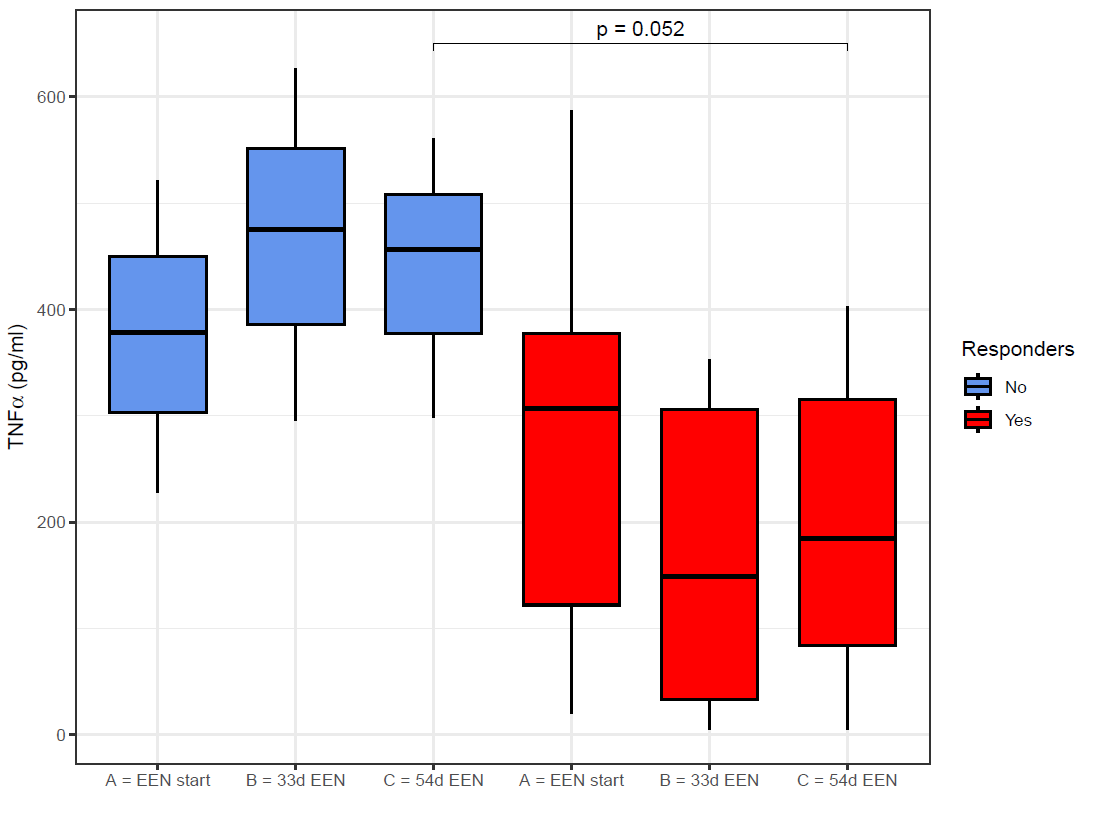


**Supplementary figure 1. TNFα concentration secreted by PBMC stimulated with the cell-free fecal filtrate of CD patients for 24 hours.**

TNFα concentration (pg/ml) secreted by PBMC stimulated with the cell-free fecal filtrate of FC non-responders (n=3; blue) and FC responders (n=8; red) at the start of EEN, after 33 days on EEN, and the end of EEN (54 days).

| **a**  **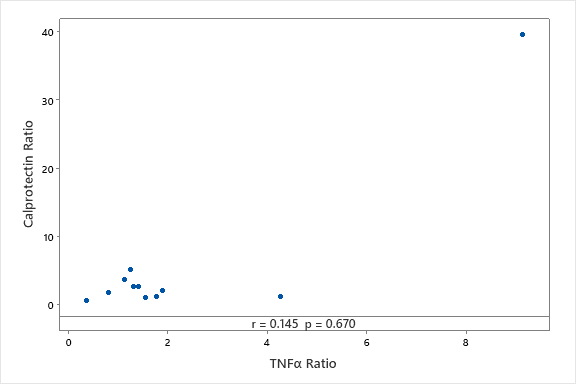** | **b**  **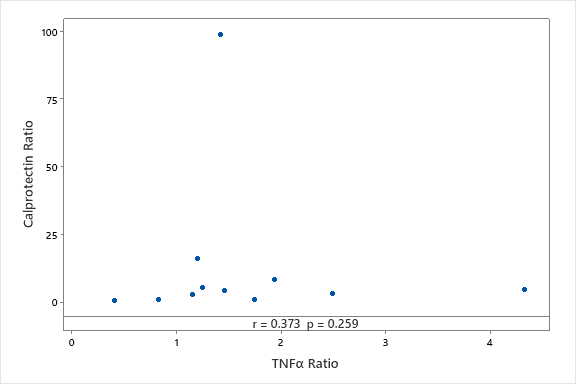** |
| --- | --- |

**Supplementary figure 2. Spearman correlations between changes in fecal calprotectin and changes in TNFα over (a) 33 days or (b) 54 days on EEN treatment in the CD cohort (n=11).**

FC non-responders (n=3; blue) and FC responders (n=8; red)


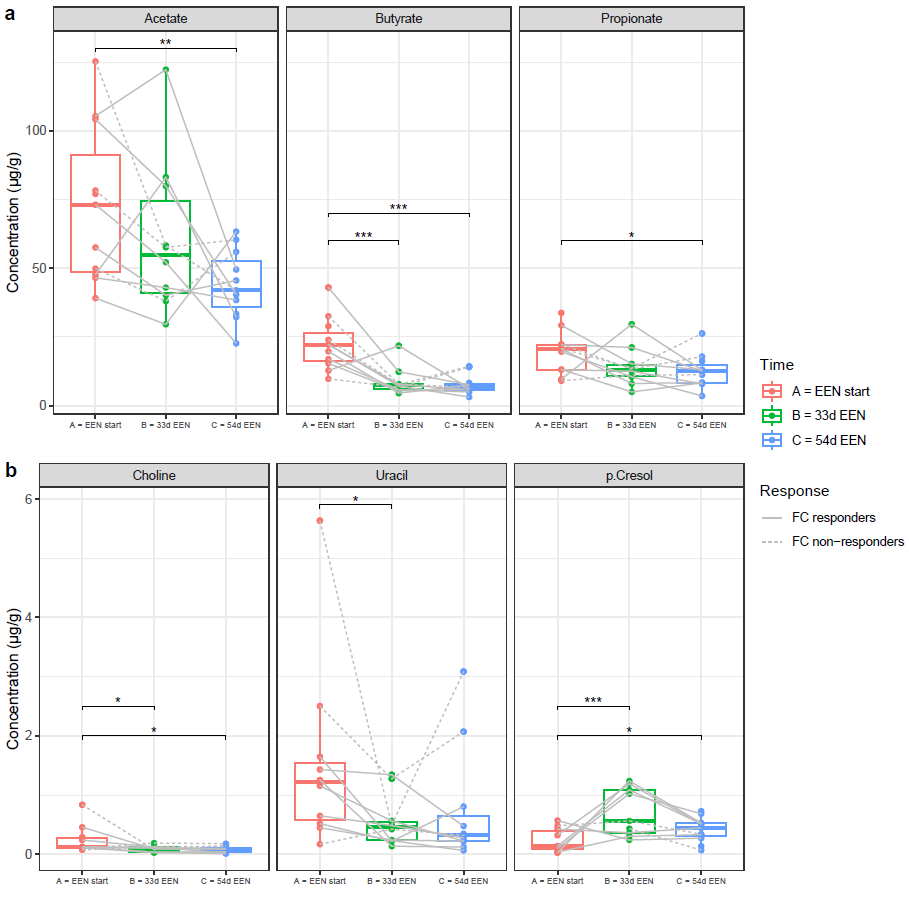


**Supplementary figure 3. Fecal metabolites significantly influenced by treatment with EEN in the full CD cohort.**

Fecal concentrations (µg/g) of (a) SCFA acetate, butyrate, and propionate and (b) choline, uracil, and p-cresol in CD patients (n=11) at the start of EEN (red), after 33 days on EEN (green) and after 54 days on EEN (blue). *p<0.05; **p<0.01; ***p<0.001.

| **a**  **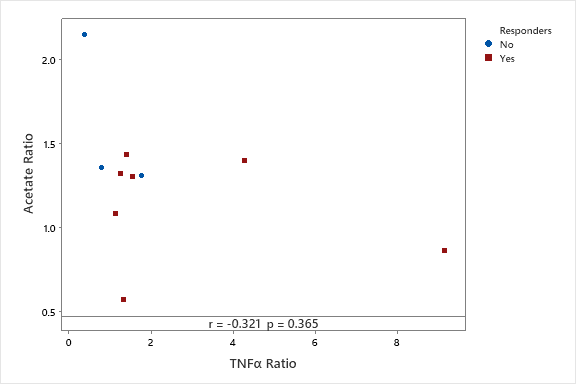** | **b**  **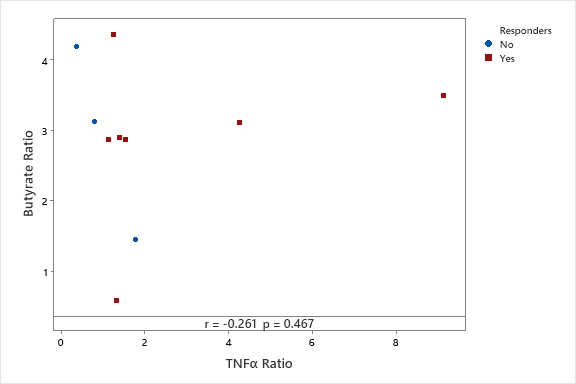** |
| --- | --- |
| **c**  **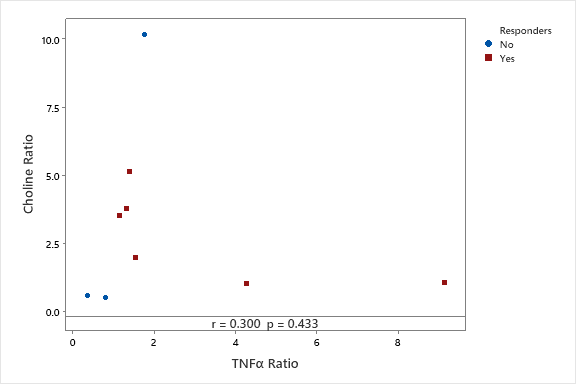** | **d**  **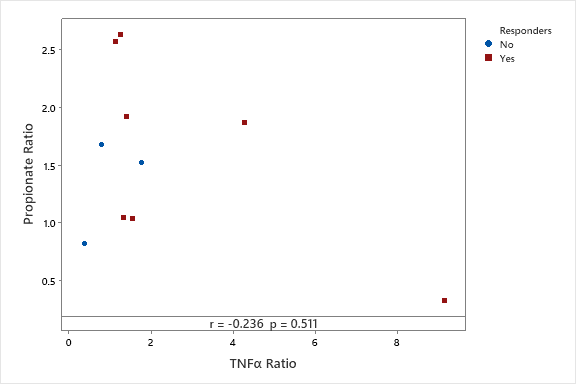** |
| **e**  **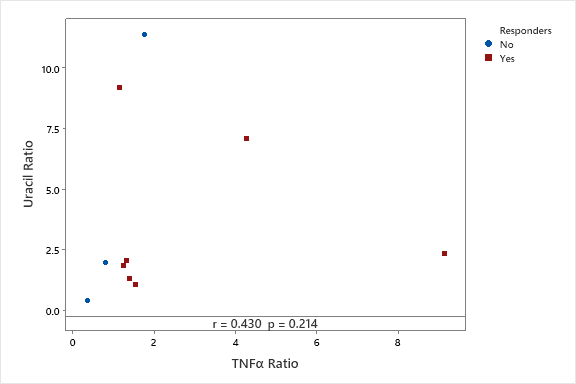** | **f**  **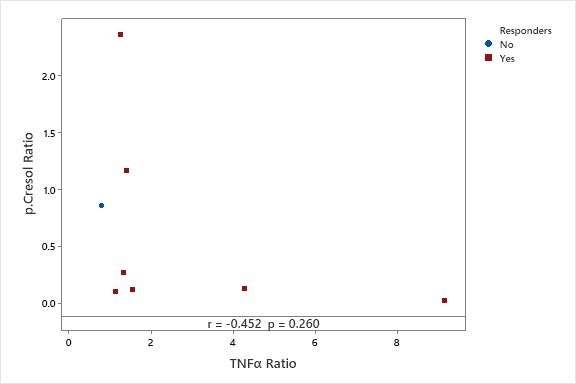** |

**Supplementary figure 4. Spearman correlations between fold-changes in TNFα and fecal metabolites during 33 days on EEN in CD patients (n=11)**

FC non-responders (n=3; blue) and FC responders (n=8; red)

| **a**  **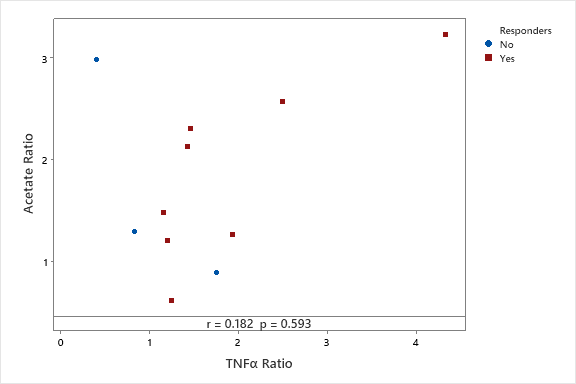** | **b**  **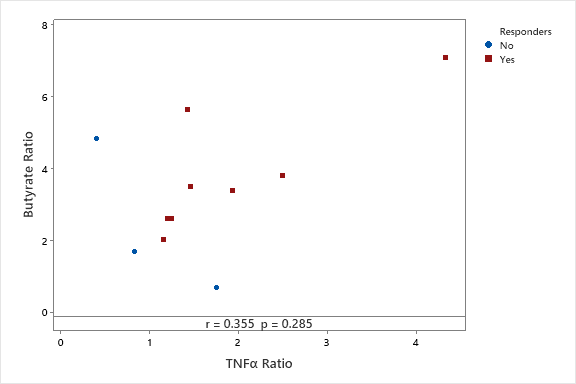** |
| --- | --- |
| **c**  **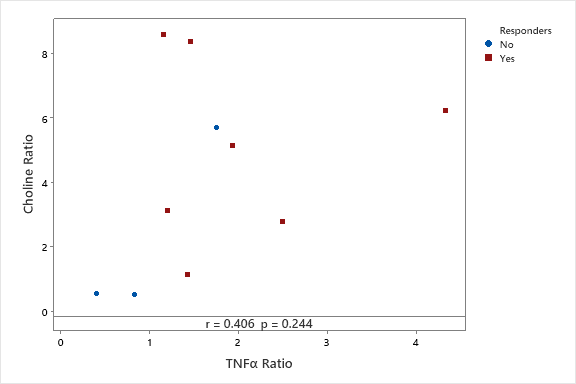** | **d**  **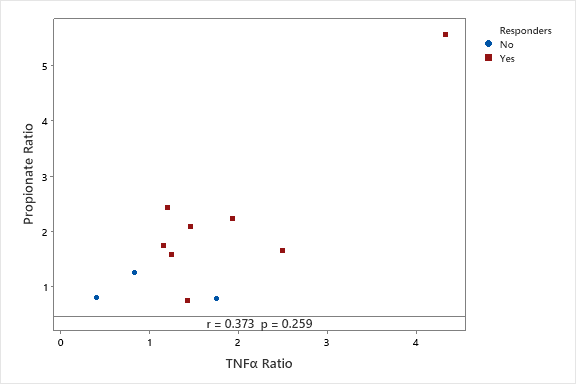** |
| **e**  **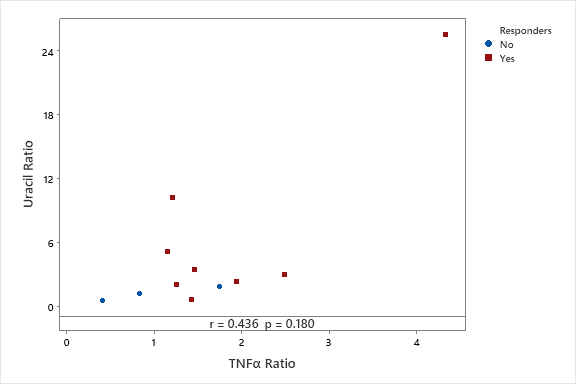** | **f**  **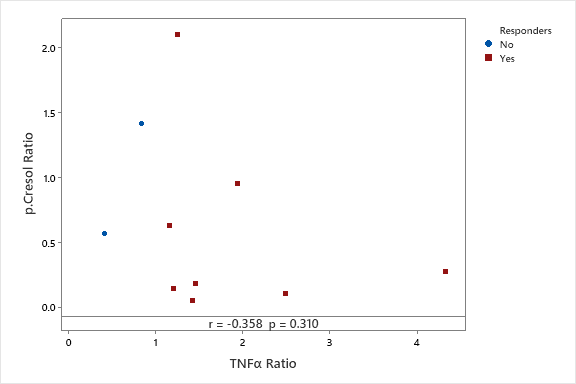** |

**Supplementary figure 5. Spearman correlations between fold-changes in TNFα and fecal metabolites during 54 days on EEN in CD patients (n=11)**

FC non-responders (n=3; blue) and FC responders (n=8; red)


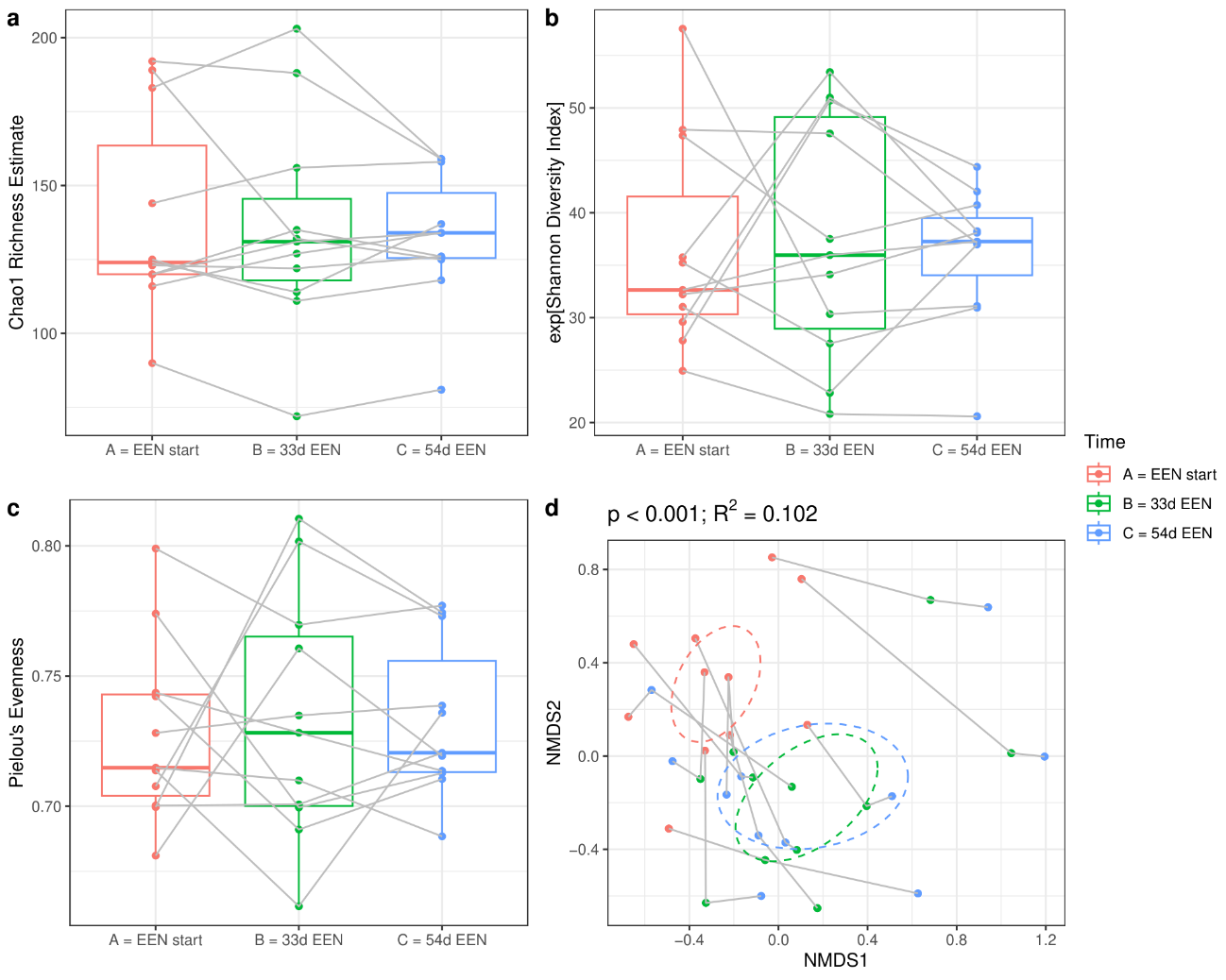


**Supplementary figure 6. Fecal microbial α-diversity and β-diversity in all CD patients prior and during treatment with EEN (n=11)**

(a) Chao1 richness estimate, (b) Shannon diversity index, (c) Pielou’s evenness, (d) non-metric multidimensional scaling (NMDS) plot in all CD patients (n=11) prior treatment (red), at 33 days on EEN (green) and 54 days on EEN (blue).

**Supplementary table 2: Mean difference (log2 fold) of microbial ASV significantly different between the start of EEN and 33 days on EEN in all CD patients.**

| **Microbial ASV** | **log2 fold change** | **p.val** | **p.adj** |
| --- | --- | --- | --- |
| ASV_27_Genus_Subdoligranulum | -11.279 | 0.006 | 0.065 |
| ASV_65_Genus_Haemophilus | -9.151 | 0.006 | 0.065 |
| ASV_9_Genus_Agathobacter | -7.515 | 0.014 | 0.075 |
| ASV_137_Genus_Monoglobus | -4.922 | 0.006 | 0.065 |
| ASV_115_Genus_Lachnospiraceae NK4A136 group | -4.896 | 0.030 | 0.114 |
| ASV_35_Genus_Bacteroides | -3.504 | 0.059 | 0.147 |
| ASV_58_Genus_Faecalibacterium | -3.096 | 0.058 | 0.147 |
| ASV_56_Species_Coprococcus.comes | -2.882 | 0.014 | 0.075 |
| ASV_61_Genus_Lachnospiraceae ND3007 group | -2.509 | 0.021 | 0.095 |
| ASV_122_Genus_Lachnoclostridium | -2.501 | 0.006 | 0.065 |
| ASV_98_Genus_Lachnospiraceae NK4A136 group | -2.493 | 0.004 | 0.065 |
| ASV_182_Genus_Lachnospiraceae FCS020 group | -2.260 | 0.006 | 0.065 |
| ASV_118_Genus_Lachnospiraceae UCG-004 | -2.171 | 0.013 | 0.075 |
| ASV_10_Species_Fusicatenibacter.saccharivorans | -2.063 | 0.014 | 0.075 |
| ASV_3_Genus_Faecalibacterium | -2.051 | 0.008 | 0.065 |
| ASV_18_Genus_Bifidobacterium | -1.661 | 0.032 | 0.114 |
| ASV_79_Genus_Lachnospiraceae NK4A136 group | -1.605 | 0.004 | 0.065 |
| ASV_2_Species_Faecalibacterium.prausnitzii | -1.600 | 0.045 | 0.136 |
| ASV_40_Species_Blautia.faecis | -1.579 | 0.018 | 0.092 |
| ASV_167_Genus_Lachnoclostridium | -1.482 | 0.007 | 0.065 |
| ASV_146_Genus_Subdoligranulum | -1.461 | 0.030 | 0.114 |
| ASV_8_Species_Faecalibacterium.prausnitzii | -1.227 | 0.056 | 0.147 |
| ASV_67_Species_Bilophila.wadsworthia | 0.842 | 0.058 | 0.147 |
| ASV_48_Species_Alistipes.finegoldii | 1.005 | 0.033 | 0.114 |
| ASV_7_Species_Blautia.massiliensis | 1.506 | 0.045 | 0.136 |
| ASV_20_Genus_[Ruminococcus] gnavus group | 1.805 | 0.053 | 0.147 |
| ASV_208_Genus_Colidextribacter | 1.814 | 0.036 | 0.114 |
| ASV_92_Species_Flavonifractor.plautii | 1.821 | 0.009 | 0.065 |
| ASV_164_Family_Oscillospiraceae | 2.013 | 0.022 | 0.095 |
| ASV_28_Species_Alistipes.putredinis | 2.418 | 0.021 | 0.095 |
| ASV_144_Genus_Lachnoclostridium | 2.507 | 0.009 | 0.065 |
| ASV_16_Genus_[Ruminococcus] torques group | 2.619 | 0.009 | 0.065 |
| ASV_220_Species_Eggerthella.lenta | 2.693 | 0.009 | 0.065 |
| ASV_33_Genus_Alistipes | 2.781 | 0.024 | 0.099 |
| ASV_214_Family_Lachnospiraceae | 2.836 | 0.036 | 0.114 |
| ASV_96_Genus_Lachnoclostridium | 2.854 | 0.009 | 0.065 |
| ASV_75_Species_Anaerostipes.hadrus | 3.097 | 0.058 | 0.147 |
| ASV_276_Genus_Incertae Sedis | 3.256 | 0.006 | 0.065 |
| ASV_128_Genus_UCG-005 | 3.563 | 0.059 | 0.147 |
| ASV_307_Genus_[Ruminococcus] torques group | 3.573 | 0.052 | 0.147 |
| ASV_175_Species_Eisenbergiella.massiliensis | 3.623 | 0.036 | 0.114 |
| ASV_139_Genus_Hungatella | 3.860 | 0.014 | 0.075 |
| ASV_73_Genus_Oscillibacter | 4.032 | 0.013 | 0.075 |
| ASV_97_Genus_UBA1819 | 5.217 | 0.007 | 0.065 |
| ASV_181_Species_Eisenbergiella.tayi | 5.253 | 0.036 | 0.114 |
| ASV_204_Genus_Faecalitalea | 6.422 | 0.022 | 0.095 |
| Log2 fold change negative: enriched at start of EEN. Log2 fold change positive: enriched at 33 days of EEN. Significance set at p.adj<0.15 | | | |

**Supplementary table 3: Mean difference (log2 fold) of microbial ASV significantly different between the start of EEN and 54 days on EEN in all CD patients.**

| **Microbial ASV** | **log2 fold change** | **p.val** | **p.adj** |
| --- | --- | --- | --- |
| ASV_9_Genus_Agathobacter | -7.437 | 0.018 | 0.113 |
| ASV_27_Genus_Subdoligranulum | -7.064 | 0.006 | 0.096 |
| ASV_137_Genus_Monoglobus | -6.458 | 0.006 | 0.096 |
| ASV_65_Genus_Haemophilus | -3.644 | 0.006 | 0.096 |
| ASV_56_Species_Coprococcus.comes | -3.133 | 0.014 | 0.097 |
| ASV_58_Genus_Faecalibacterium | -2.831 | 0.044 | 0.143 |
| ASV_122_Genus_Lachnoclostridium | -2.475 | 0.006 | 0.096 |
| ASV_18_Genus_Bifidobacterium | -2.166 | 0.014 | 0.097 |
| ASV_115_Genus_Lachnospiraceae NK4A136 group | -2.096 | 0.042 | 0.143 |
| ASV_98_Genus_Lachnospiraceae NK4A136 group | -2.012 | 0.004 | 0.096 |
| ASV_3_Genus_Faecalibacterium | -2.010 | 0.011 | 0.097 |
| ASV_118_Genus_Lachnospiraceae UCG-004 | -1.976 | 0.009 | 0.097 |
| ASV_163_Genus_Dorea | -1.861 | 0.021 | 0.116 |
| ASV_10_Species_Fusicatenibacter.saccharivorans | -1.834 | 0.011 | 0.097 |
| ASV_107_Genus_UCG-002 | -1.436 | 0.035 | 0.137 |
| ASV_79_Genus_Lachnospiraceae NK4A136 group | -1.233 | 0.005 | 0.096 |
| ASV_167_Genus_Lachnoclostridium | -0.964 | 0.019 | 0.114 |
| ASV_38_Species_Odoribacter.splanchnicus | 1.005 | 0.044 | 0.143 |
| ASV_92_Species_Flavonifractor.plautii | 1.300 | 0.023 | 0.116 |
| ASV_20_Genus_[Ruminococcus] gnavus group | 1.550 | 0.032 | 0.137 |
| ASV_75_Species_Anaerostipes.hadrus | 1.718 | 0.044 | 0.143 |
| ASV_28_Species_Alistipes.putredinis | 2.197 | 0.030 | 0.137 |
| ASV_164_Family_Oscillospiraceae | 2.493 | 0.014 | 0.097 |
| ASV_16_Genus_[Ruminococcus] torques group | 2.519 | 0.005 | 0.096 |
| ASV_214_Family_Lachnospiraceae | 2.622 | 0.036 | 0.137 |
| ASV_585_Genus_Colidextribacter | 2.679 | 0.030 | 0.137 |
| ASV_220_Species_Eggerthella.lenta | 2.837 | 0.014 | 0.097 |
| ASV_276_Genus_Incertae Sedis | 3.292 | 0.009 | 0.097 |
| ASV_227_Species_Hungatella.hathewayi | 3.361 | 0.022 | 0.116 |
| ASV_73_Genus_Oscillibacter | 3.510 | 0.044 | 0.143 |
| ASV_139_Genus_Hungatella | 3.824 | 0.022 | 0.116 |
| ASV_307_Genus_[Ruminococcus] torques group | 4.140 | 0.035 | 0.137 |
| ASV_97_Genus_UBA1819 | 5.386 | 0.008 | 0.097 |
| ASV_204_Genus_Faecalitalea | 7.041 | 0.014 | 0.097 |
| ASV_82_Species_Blautia.caecimuris | 7.991 | 0.036 | 0.137 |
| Log2 fold change negative: enriched at start of EEN. Log2 fold change positive: enriched at 54 days of EEN. Significance set at p.adj<0.15 | | | |

**Supplementary table 4: Mean difference (log2 fold) of microbial ASV significantly different between the start of EEN and 33 days on EEN in FC responders.**

| **Microbial ASV** | **log2 fold change** | **p.val** | **p.adj** |
| --- | --- | --- | --- |
| ASV_9_Genus_Agathobacter | -8.682 | 0.036 | 0.147 |
| ASV_27_Genus_Subdoligranulum | -8.184 | 0.022 | 0.147 |
| ASV_65_Genus_Haemophilus | -6.342 | 0.022 | 0.147 |
| ASV_137_Genus_Monoglobus | -6.041 | 0.022 | 0.147 |
| ASV_101_Genus_Lachnospiraceae UCG-004 | -5.910 | 0.022 | 0.147 |
| ASV_118_Genus_Lachnospiraceae UCG-004 | -5.111 | 0.036 | 0.147 |
| ASV_58_Genus_Faecalibacterium | -4.656 | 0.035 | 0.147 |
| ASV_24_Genus_Subdoligranulum | -3.386 | 0.021 | 0.147 |
| ASV_182_Genus_Lachnospiraceae FCS020 group | -3.120 | 0.022 | 0.147 |
| ASV_122_Genus_Lachnoclostridium | -3.004 | 0.014 | 0.147 |
| ASV_98_Genus_Lachnospiraceae NK4A136 group | -2.900 | 0.014 | 0.147 |
| ASV_56_Species_Coprococcus.comes | -2.837 | 0.036 | 0.147 |
| ASV_3_Genus_Faecalibacterium | -2.576 | 0.035 | 0.147 |
| ASV_10_Species_Fusicatenibacter.saccharivorans | -2.466 | 0.021 | 0.147 |
| ASV_167_Genus_Lachnoclostridium | -2.380 | 0.030 | 0.147 |
| ASV_8_Species_Faecalibacterium.prausnitzii | -2.108 | 0.014 | 0.147 |
| ASV_40_Species_Blautia.faecis | -1.705 | 0.030 | 0.147 |
| ASV_79_Genus_Lachnospiraceae NK4A136 group | -1.517 | 0.014 | 0.147 |
| ASV_92_Species_Flavonifractor.plautii | 2.003 | 0.014 | 0.147 |
| ASV_144_Genus_Lachnoclostridium | 2.275 | 0.036 | 0.147 |
| ASV_16_Genus_[Ruminococcus] torques group | 2.564 | 0.021 | 0.147 |
| ASV_220_Species_Eggerthella.lenta | 2.583 | 0.022 | 0.147 |
| ASV_96_Genus_Lachnoclostridium | 2.856 | 0.022 | 0.147 |
| ASV_276_Genus_Incertae Sedis | 3.039 | 0.014 | 0.147 |
| ASV_139_Genus_Hungatella | 3.692 | 0.036 | 0.147 |
| ASV_73_Genus_Oscillibacter | 3.982 | 0.036 | 0.147 |
| ASV_181_Species_Eisenbergiella.tayi | 4.375 | 0.036 | 0.147 |
| ASV_97_Genus_UBA1819 | 5.131 | 0.014 | 0.147 |
| Log2 fold change negative: enriched at start of EEN. Log2 fold change positive: enriched at 33 days of EEN. Significance set at p.adj<0.15 | | | |

**Supplementary table 5: Mean difference (log2 fold) of microbial ASV significantly different between the start of EEN and 54 days on EEN in FC responders.**

| **Microbial ASV** | **log2 fold change** | **p.val** | **p.adj** |
| --- | --- | --- | --- |
| ASV_137_Genus_Monoglobus | -6.702 | 0.022 | 0.128 |
| ASV_65_Genus_Haemophilus | -6.558 | 0.022 | 0.128 |
| ASV_27_Genus_Subdoligranulum | -6.014 | 0.022 | 0.128 |
| ASV_182_Genus_Lachnospiraceae FCS020 group | -4.723 | 0.022 | 0.128 |
| ASV_24_Genus_Subdoligranulum | -4.512 | 0.014 | 0.128 |
| ASV_58_Genus_Faecalibacterium | -4.126 | 0.022 | 0.128 |
| ASV_122_Genus_Lachnoclostridium | -3.322 | 0.014 | 0.128 |
| ASV_101_Genus_Lachnospiraceae UCG-004 | -3.059 | 0.022 | 0.128 |
| ASV_3_Genus_Faecalibacterium | -2.850 | 0.022 | 0.128 |
| ASV_98_Genus_Lachnospiraceae NK4A136 group | -2.194 | 0.014 | 0.128 |
| ASV_10_Species_Fusicatenibacter.saccharivorans | -1.954 | 0.021 | 0.128 |
| ASV_167_Genus_Lachnoclostridium | -1.577 | 0.022 | 0.128 |
| ASV_123_Genus_Lachnoclostridium | -1.066 | 0.022 | 0.128 |
| ASV_79_Genus_Lachnospiraceae NK4A136 group | -1.061 | 0.021 | 0.128 |
| ASV_92_Species_Flavonifractor.plautii | 1.584 | 0.014 | 0.128 |
| ASV_16_Genus_[Ruminococcus] torques group | 2.563 | 0.014 | 0.128 |
| ASV_220_Species_Eggerthella.lenta | 2.748 | 0.022 | 0.128 |
| ASV_276_Genus_Incertae Sedis | 3.177 | 0.014 | 0.128 |
| ASV_139_Genus_Hungatella | 3.711 | 0.022 | 0.128 |
| ASV_97_Genus_UBA1819 | 5.313 | 0.014 | 0.128 |
| Log2 fold change negative: enriched at start of EEN. Log2 fold change positive: enriched at 54 days of EEN. Significance set at p.adj<0.15 | | | |

**
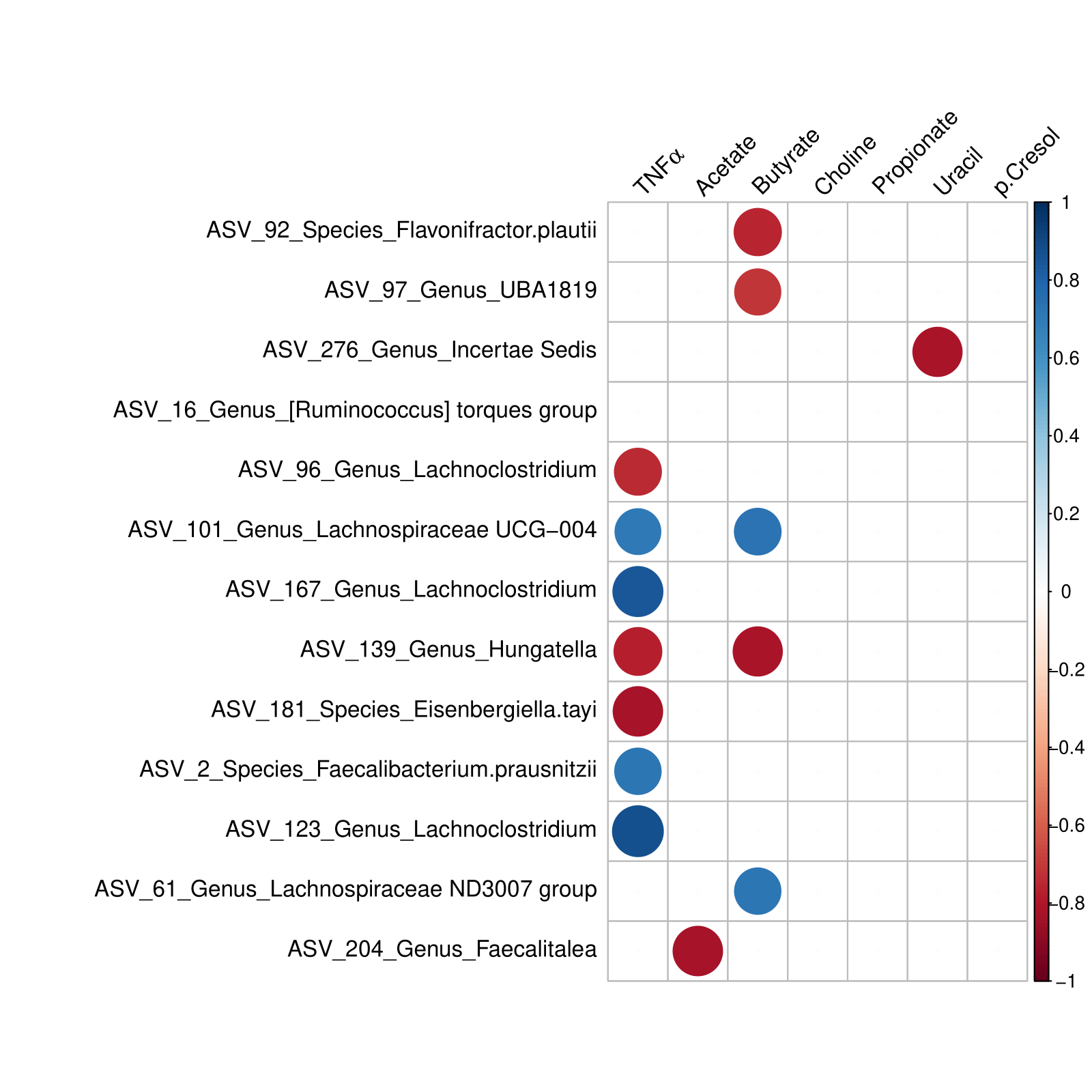
**

**Supplementary figure 7. Heatmap displaying spearman correlations between microbial ASVs, secreted TNFα and fecal metabolites after 54 days on EEN in the CD cohort (n=11)**

White squares indicate non-significant correlations between microbial ASVs and metabolites.
